# Is Pulmonary Artery Pulsatility Index (PAPi) a Predictor of Outcome after Pulmonary Endarterectomy?

**DOI:** 10.3390/jcm11154353

**Published:** 2022-07-27

**Authors:** Sofia Martin-Suarez, Gregorio Gliozzi, Giulio Giovanni Cavalli, Valentina Orioli, Antonio Loforte, Saverio Pastore, Barbara Rossi, Davide Zardin, Nazzareno Galiè, Massimiliano Palazzini, Fabio Dardi, Francesco Saia, Fabio Niro, Davide Pacini

**Affiliations:** 1 Cardiac Surgery Unit, Cardio Thoracic and Vascular Department, S. Orsola Hospital IRCCS, Bologna University, 40138 Bologna, Italy; greg.gliozzi@hotmail.it (G.G.); giuliogiovannicavalli@gmail.com (G.G.C.); valentina.orioli4@studio.unibo.it (V.O.); antonino.loforte@aosp.bo.it (A.L.); davide.pacini@unibo.it (D.P.); 2 Cardiac Anaesthesia Unit, Cardio Thoracic and Vascular Department, S. Orsola Hospital IRCCS, Bologna University, 40138 Bologna, Italy; saverio.pastore@aosp.bo.it (S.P.); barbara.rossi@aosp.bo.it (B.R.); 3 Cardiopulmonary Perfusion Service, Cardiac Surgery Unit, Cardio Thoracic and Vascular Department, S. Orsola Hospital IRCCS, Bologna University, 40138 Bologna, Italy; davide.zardin@libero.it; 4 Cardiology Unit, Cardio Thoracic and Vascular Department, S. Orsola Hospital IRCCS, Bologna University, 40126 Bologna, Italy; nazzareno.galie@unibo.it (N.G.); massimiliano.palazzini@unibo.it (M.P.); fabio.dardi@unibo.it (F.D.); francesco.saia@aosp.bo.it (F.S.); 5 Cardiovascular Radiology Unit, Cardio Thoracic and Vascular Department, S. Orsola Hospital IRCCS, Bologna University, 40126 Bologna, Italy; fabio.niro@aosp.bo.it

**Keywords:** chronic thromboembolic pulmonary hypertension, pulmonary endarterectomy, right ventricular failure, pulmonary artery pulsatility index

## Abstract

Background: Pulmonary endarterectomy (PEA) is the gold standard therapy for chronic thromboembolic pulmonary hypertension (CTEPH). Traditionally, pulmonary vascular resistance (PVR) represents the main prognostic factor after surgery. The pulmonary artery pulsatility index (PAPi) has been proposed for the assessment of RV in advanced heart failure, but it has never been applied in CTEPH patients. The aim of the present study is to describe PAPi in patients who underwent PEA, before and after surgery, and to define its predictive impact on postoperative outcomes. Methods: We retrospectively reviewed 188 consecutive adult patients who underwent PEA, between December 2003 and December 2021. PAPi was calculated for 186 patients and reported. Patients were partitioned in two groups using median preoperative PAPi as cutoff value: Group 1 with PAPi ≤ 8.6 (*n* = 94) and Group 2 with PAPi > 8.6 (*n* = 92). The propensity-score-matched analysis identified 67 pairs: Early outcomes were compared between two groups. Results: Mean preoperative PAPi was 10.3 ± 7.2. Considering matched populations, no differences emerged in terms of postoperative hemodynamics; Group 1 demonstrated higher 90-day mortality significance (10.4% vs. 3.0%, *p* = 0.082); the need for mechanical circulatory support (MCS) was similar, but successful weaning was unlikely (25% vs. 85.7%, *p* = 0.032). Conclusions: Mean PAPi in the CTEPH population is higher than in other diseases. Low PAPi (≤8.6) seems to be associated with lower postoperative survival and successful weaning from MCS.

## 1. Introduction

Chronic thromboembolic pulmonary hypertension (CTEPH) is a type of chronic and evolutive pulmonary hypertension (PH) secondary to the thrombotic obstruction of the pulmonary arteries, due to embolism and/or in situ thrombosis. The obstruction of the pulmonary arteries, occurring at different levels, from lobar to subsegmental arteries together with the vascular vasoconstriction, promotes vascular remodeling. The evolution of the disease involves a progressive increase in pulmonary pressure and pulmonary vascular resistance (PVR), which is the right ventricle (RV) afterload. In CTEPH, the RV turns hypertrophic and dilated, leading to RV dysfunction and failure.

Pulmonary endarterectomy (PEA) is the gold standard surgical treatment for CTEPH: It requires experienced surgeons with strong technical skills and knowledge, as well as a dedicated PH team involving cardiologists, anesthesiologists, and radiologists. Moreover, postoperative outcomes (hemodynamics improvement, mortality, and morbidity) depend on the moment in the natural history of the disease in which it is performed. In cases of advanced disease, surgery may not be completely resolutive and could be burdened by an increased perioperative risk.

Different degrees of PH, mainly due to the development of small artery disease in non-obstructed segments, could determine different degrees of RV dysfunction. Postoperative RV dysfunction occurs approximately in one-third of patients and is an important determinant of complications and risk of death. Traditionally, PVR is the main prognostic factor after PEA, but the anatomical and hemodynamic evaluation of the quality of the pulmonary circulation should be completed with a careful assessment of the degree of dysfunction and resilience of the RV [1,2,3].

Many parameters of RV function have been proposed since its oblique contraction and geometry make its assessment harder. The pulmonary artery pulsatility index (PAPi), defined as the ratio between pulmonary artery pulse pressure and central venous pressure, has been shown to be a predictive factor of RV dysfunction and outcomes in different clinical settings: from candidates to the implantation of left ventricular assist devices (LVADs), to elective cardiac surgery population [4,5,6,7]. Potentially, the evaluation of RV function could be crucial, allowing the appraisal of the risks of PEA intervention, and the estimation of the odds of recovery of ventricular function as a surrogate of the degree of recovery from pulmonary hypertension and its clinical benefit.

The aim of the study is to describe PAPi in patients with CTEPH candidates to PEA operation and its relationship with other hemodynamic parameters, as well as its trend after surgery. We also hypothesize that it might predict postoperative mortality, RV dysfunction requiring mechanical circulatory support (MCS), and pulmonary hemodynamics. To this end, we retrospectively analyzed a cohort of 188 PEA-operated patients.

## 2. Material and Methods

Between December 2003 and December 2021, 188 consecutive adult patients (>17 years) underwent PEA. Our series was retrospectively reviewed: Baseline features (with a particular focus on pulmonary hemodynamics), and operative and postoperative data were collected. For each patient, PAPi was calculated accordingly with the following equation:

$$\mathrm{PAPi}=\frac{SystolicPAP\left(\mathrm{mmHg}\right)-DiastolicPAP\left(\mathrm{mmHg}\right)}{RAP\left(\mathrm{mmHg}\right)}$$

Two patients were excluded from the analysis because of the incompleteness of preoperative right heart catheterization (RHC) data and the impossibility to calculate PAPi. In order to describe the PAPi across the entire series, hemodynamic data were first reported in quartiles: quartile 1 (Q1, *n* = 46) with the lowest mean PAPi; quartile 2 (Q2, *n* = 47) and quartile 3 (Q3, *n* = 47) with PAPi values near to median; and quartile 4 (Q4, *n* = 46) with the highest PAPi.

Our cohort was partitioned into two subgroups using the median PAPi as a cutoff: The first group included patients with lower PAPi (≤8.6; *n* = 94), while the second group had higher PAPi (>8.6; *n* = 92). In order to reduce selection bias and to reduce the burden of confounding factors (with particular consideration given to pulmonary vascular resistance), a propensity score (PS) matching was performed with the 1:1 nearest neighbor method and the matching tolerance set at 0.1.

**Figure 1:**
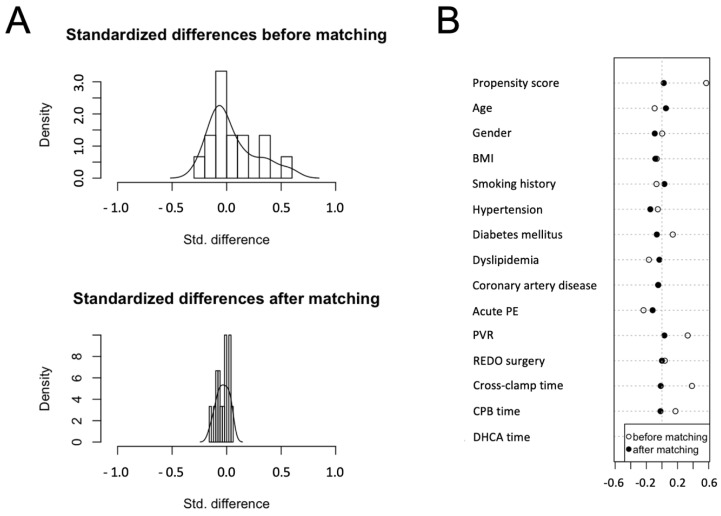
Propensity score (PS) matching: the graphs show the distribution of standardized differences before and after matching (**A**) and the baseline features chosen as matching variables (**B**).

PAPi ≤ 8.6 was used as a dependent variable, and matching variables were chosen considering risk factors for early mortality traditionally reported in the literature and considering a preliminary logistic regression for 90-day mortality: age, gender, body mass index (BMI), smoking history, hypertension, diabetes mellitus, dyslipidemia, coronary artery disease, history of acute pulmonary embolism, PVR, REDO surgery, cardiopulmonary bypass (CPB) time, cross-clamp time, and deep hypothermic circulatory arrest time (DHCA). As result, 67 matched pairs were obtained for the final analysis. Standardized differences before and after matching are reported in Figure 1.

Postoperative outcomes were compared between two groups, for which 90-day mortality and the need for postcardiotomy extracorporeal life support (ECLS) were considered as the primary endpoint.

Dichotomic variables were expressed as numbers and percentages and were compared using the χ2 test or Fisher’s exact test, when appropriate; continuous variables are expressed as mean ± standard deviation, and they were compared using Student’s *t*-test.

Risk factor analysis for primary endpoints was performed through a binary logistic regression model.

Early survival was analyzed using the Kaplan–Meier method and compared through the log-rank test.

A *p*-value < 0.05 was considered statistically significant in all analyses.

All statistical analyses were performed with IBM SPSS software v.26 (Chicago, IL, USA).

## 3. Results

### 3.1. Baseline Features

The mean overall preoperative PAPi was 10.3 ± 7.2. The inference between groups divided based on the PAPi median value (≤8.6 vs. >8.6) revealed no large differences, not even when matched. The mean PAPi value was strongly reduced (5.6 ± 2.0) in the first group, while in the second one, the mean value was 15 ± 7.4, maintaining this strong difference distribution after matching (5.8 ± 1.9 vs. 15 ± 7.9, *p* = 0.000). Hemodynamics and echocardiographic parameters in different PAPi quartiles are reported in Table 1: systolic pulmonary artery pressure (PAPs) was similar in all quartiles (*p* = 0.815), while diastolic pulmonary artery pressure (PAPd) and right atrial pressure (RAP) gradually decreased across quartiles (*p* = 0.000); there was a significant difference between groups in terms of PVR (*p* = 0.006).

The mean age was 58 ± 15 years, the majority of patients were female (63.4%), and the mean BMI was 26.1 ± 4.9 kg/m^2^. Less than 50% of patients had typical cardiovascular risk factors (4% had diabetes mellitus, 47% had systemic arterial hypertension, 33% had dyslipidemia, and 32% had a history of smoking). From 66% to 70.7% of patients were on specific pulmonary vasodilator therapy before the operation.

Considering the lung function, even without reaching the statistical significance, patients with a higher PAPi had a higher incidence of obstructive profile at the spirometry (41.3% vs. 27.7% and 41.8 vs. 25.4 in the matched analysis, *p* < 0.05). Previous acute pulmonary embolism was documented in 66.3% of the group with PAPi > 8.6, compared with 54.3% in the lower PAPi group (*p* = 0.75). Interestingly, predisposing factors for CTEPH, such as previous history of cancer (38.8% vs. 25.4%, *p* = 0.10) or thrombophilia (40.3% vs. 23.9%, *p* < 0.05), were more frequent in the higher PAPi population. Analyzing the level of obstruction of the thromboembolic disease (main lobar, segmental, subsegmental arteries) at the CT angiography scan, higher PAPi patients had a main lobar arteries involvement in 70% of cases vs. 59.7% in lower PAPi without statistical significance (*p*= 0.20), while the subsegmental arteries were obstructed or stenotic in 64% of lower PAPi group vs. 46.3% when matched (*p* < 0.05).

Considering echocardiographic data, the most striking data which reflected the correlation of the PAPi value with the performance of the right ventricle were the right ventricular shortening fraction (RVSF) and the degree of tricuspid regurgitation (TR). In lower PAPi group, RVSF resulted in significantly lower values (24 ± 9 vs. 30 ± 10%, *p* < 0.01) and the percentage of patients with severe TR was 35% vs. 19.6% (*p* = 0.03). All quartiles demonstrated a good mean left ventricular ejection fraction (LVEF), while the right ventricular shortening fraction (RVEF) increased across quartiles (*p* < 0.01).

These functional aspects are consistent with hemodynamic data found in RHC. RAP values were higher in the low-PAPi group (11 ± 5 vs. 5 ± 2 mmHg *p* < 0.01), whereas cardiac index (CI) was lower (2.4 ± 0.7 vs. 2.8 ± 0.8 l/min/m^2^ *p* < 0.01). Lower PAPi also correlated with higher PVR (9.9 ± 4.2 vs. 8.5 ± 3.1 Wood units (WU), *p* = 0.01), while CO (4.4 vs. 5.0 l/min) and CI (2.4 vs. 2.8) values were significantly lower (*p* < 0.05).

Elastic characteristics of the pulmonary artery system are reflected by the PAPd, one factor of the denominator of the PAPi formula that was clearly higher in the low PAPi group (29 ± 12 vs. 23 ± 7 mmHg, *p* < 0.01). Baseline features are reported in Table 2.

### 3.2. Early Outcomes

Early outcomes are reported in Table 3.

Overall mean CPB and cross-clamp time were 284 ± 69 and 160 ± 47 min, respectively. DHCA was necessary in 74.2% of cases, with a mean DHCA time of 30 ± 17 min.

Hemodynamics measured during the first postoperative day showed a mean PAP (PAPm) of 25 ± 7 mmHg and a mean PVR of 4.1 ± 2.9 UW. After surgery, PAPi significantly decreased to 3.8 + 2.6 (*p* = 0.000).

The overall 90-day mortality was 9.6%, with a significant decrease in the second part of our series (14.6% vs. 4.3%, *p* = 0.017). The need for postoperative ECLS was 12.9%; re-thoracotomy for bleeding occurred in 13.4% of cases, permanent neurological disease in 1.1%, and permanent renal replacement therapy in 3.2%.

Considering the matched population, no differences emerged in terms of postoperative hemodynamics (*p* > 0.05). Notably, different preoperative PAPi demonstrated different 90-day mortality without reaching statistical significance (10.4% vs. 3.0%, *p* = 0.082); the need for ECLS was similar, but successful weaning was performed in only 25% of patients with low preoperative PAPi vs. 85.7% of patients with high PAPi (*p* = 0.032).

**Figure 2:**
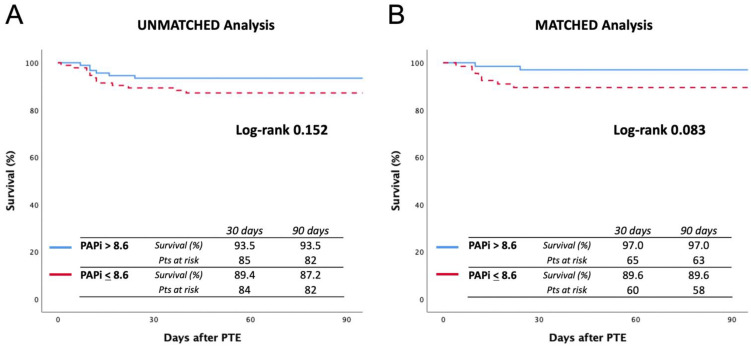
Ninety-day survival curves before (**A**) and after (**B**) matching. The cutoff value was considered a PAPi score of 8.6.

Survival curves are reported in Figure 2. The unmatched analysis showed a 90-day survival of 87.2% in the low-PAPi group vs. 93.5% in high-PAPi patients (log-rank 0.152), while in the matched analysis, 90-day survival was 89.6% vs. 97.0% (log-rank 0.083), respectively.

**Figure 3:**
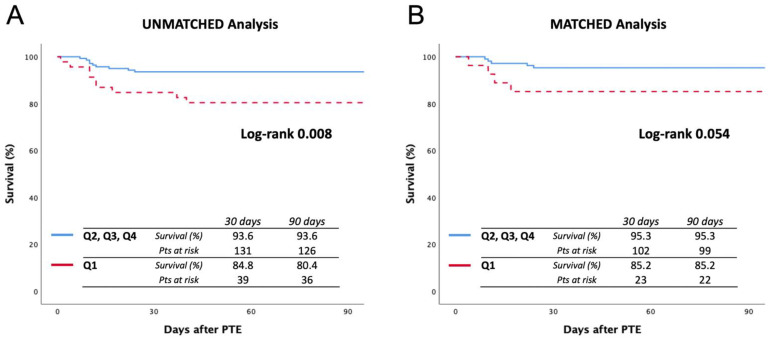
Ninety-day survival curves before (**A**) and after (**B**) matching, comparing quartile 1 vs. quartiles 2, 3, and 4. Quartile 1 includes a PAPi value lower than 5.

In addition, the comparison of survival in terms of PAPi quartiles is reported in Figure 3. Specifically, Q1 was compared with the remaining population: In the unmatched analysis, the 90-day survival rates were 80.4% and 93.6%, respectively (log-rank 0.008), while in the matched analysis, 90-day survival rates were 85.2% and 95.3%, respectively (log-rank 0.054).

Logistic regression for 90-day mortality in matched cohort PAPi (as continuous variable) did not emerge as a predictive factor (*p* = 0.538, OR 0.96), but PAPi ≤ 8.6 (*p* = 0.105, OR 3.8, CI 95% 0.76–18.9) and Q1 (*p* = 0.074, OR 3.55, CI 95% 0.88–14.3) demonstrated a trend toward significance.

## 4. Limitations

The present study is prone to well-known limitations due to its retrospective nature. Previously, PAPi has not been used in a population completely affected by pulmonary hypertension; therefore, the use of median PAPi was a statistical stratagem to study this cohort, in absence of landmarks or reference points. Confounding factors were adjusted through PS matching, but the final groups were relatively small. Moreover, the series covered a long period of time, during which some changes in surgical techniques have been made.

## 5. Discussion

PEA has been defined as the gold standard treatment for CTEPH. Although it remains the best and potentially curative treatment, improvements in our knowledge of natural history, pathophysiology, and outcomes allow us to affirm that, in a significant proportion of patients, the pressure in the pulmonary circulation remains altered after surgery. Furthermore, PEA itself is characterized by CPB, with a period of DHCA, which involves a higher risk of complications than standard cardiac surgery. For these reasons, before surgery, it is essential to perform a careful selection of candidates and a better stratification of surgical risk [8].

The clinical phenotype of CTEPH is very heterogenous: from patients with severe PH, with distal occlusive pathology, and presumably with severe involvement of pulmonary microvasculature, to patients with important proximal lesions and almost no PH. RV dysfunction with or without tricuspid valve involvement, or the degree of lung parenchymal damage, is also extremely variable.

In CTEPH, PVR is the main predictor of postoperative morbidity and mortality, while lesions’ extent (proximal versus distal) only affects surgical feasibility and operative times [9].

Several authors have sought to identify other risk factors and scores that allow identifying those patients at risk of postoperative outcomes, such as mortality, need for ECLS, and residual PH. In 2021, Ghio et al. reported an excellent retrospective analysis, identifying an extremely useful risk model that considers PVR, pulmonary artery compliance (CPA), and PaO2 [7]. The derived score model allowed the identification of a subgroup at low risk of clinical worsening at the same level of that observed in patients without residual PH. The constant relationship between PVR and CPA has important clinical applications. It has been suggested that a substantial decline in CPA occurs before increases in PVR [10]. Thus, assessment of CPA may allow for diagnosis of pulmonary vascular disease before PVR elevations. Resistance and compliance in the pulmonary circulation exhibit an inverse hyperbolic relationship. The product of resistance and compliance (RC time) is mostly constant [11,12]. Patients with the proximal chronic thromboembolic pulmonary vascular disease also have reduced RC time, compared with idiopathic PH or distal CTEPH [13]. This is caused by increased wave reflection from proximal obstructions. Similar findings were observed in experimental animal models of proximal and distal CTEPH.

The PAPi value, which represents the interaction between RV function and arterial pulmonary vasculature, has been previously associated with RV failure in patients with acute inferior myocardial infarction requiring temporary MCS as well after LVAD implantation [5,6,7].

Thus, PAPi is probably the only parameter that describes the relationship between pulmonary compliance and RV contractility and, consequently, possibly one of the main determinants of postoperative prognosis and survival [14].

The usefulness of PAPi as a predictor of mortality in patients with PAH has been tested by Mazimba et al. PAPi was employed in the risk stratification of patients with PAH. From a clinical utility standpoint, PAPi is easy to calculate and relies on only three invasively measured parameters; on the other hand, it gives at once a considerable amount of information about filing status (RAP), tension, and compliance of pulmonary vascular compartment, reflecting the status of the entire cardiopulmonary unit [14]. They demonstrated that decreased PAPi was independently associated with mortality during the five years of follow-up and that patients in the lowest PAPi quartile had a profile of hemodynamic indices associated with severe RV failure in patients with PAH.

PAPi has never been used or described in a CTEPH population: The mean PAPi value in this cohort was typically higher and more variable than that in other diseases, reflecting, once more, the clinical heterogeneity of CTEPH patients. Lower PAPi scores seemed to be associated with higher PVR, reduced CI, and reduced shortening fraction, signs of a more compromised ventricular–pulmonary unit.

Furthermore, we sought to identify whether these findings could have an impact on postoperative results. According to our results, at equal preoperative conditions (notably, PVR), patients with low PAPi showed worse 90-day mortality and more difficult weaning from ECLS. No differences were noted in terms of early hemodynamic results.

In other words, RV dysfunction expressed with PAPi seems to have an impact on morbidity and mortality after PEA but does not affect the technical success of the operation, with good hemodynamic results.

The present study on the impact of the PAPi needs to be further investigated and eventually validated in larger PEA registries and/or extended to a global CTEPH population, perhaps including patients considered not operable. We believe that this parameter, routinely used in other clinical areas, can be an interesting tool in the preoperative risk stratification of CTEPH patients.

## 6. Conclusions

In CTEPH, PAPi is higher and more heterogeneous than in other clinical conditions, such as advanced heart failure. PAPi seems to be correlated to more compromised preoperative hemodynamic conditions, with higher rates of PVR and RV dysfunction. Patients with lower PAPi showed a lower postoperative survival rate and harder weaning from ECLS.

More studies are needed in order to validate these preliminary findings.

## Figures and Tables

**Table 1:** Preoperative hemodynamic and echocardiographic parameters in different PAPi quartiles. PAPi, Pulmonary Artery Pulsatility index; PAPs, systolic pulmonary artery pressure; PAPd, diastolic pulmonary artery pressure; PAPm, mean pulmonary artery pressure; PCWP, pulmonary capillary wedge pressure; RAP, right atrial pressure; TPG, transpulmonary gradient; CO, cardiac output; CI, Cardiac Index; PVR, pulmonary vascular resistance; LVEF, left ventricular ejection fraction; RVSF, right ventricular shortening fraction; RVEF, right ventricular ejection fraction; RV, right ventricular.

	Quartile 1 (*n* = 46)	Quartile 2 (*n* = 47)	Quartile 3 (*n* = 47)	Quartile 4 (*n* = 46)	
	Mean ± SD	Mean ± SD	Mean ± SD	Mean ± SD	*p*-Value
PAP Index	4 ± 1	7 ± 1	10 ± 1	20 ± 8	**0.000**
Right heart catheterization					
PAPs, mmHg	84 ± 18	84 ± 18	85 ± 15	87 ± 22	0.815
PAPd, mmHg	32 ± 13	25 ± 8	25 ± 7	22 ± 7	**0.000**
PAPm, mmHg	52 ± 14	47 ± 11	48 ± 9	47 ± 12	0.136
PCWP, mmHg	12 ± 7	10 ± 3	9 ± 2	8 ± 3	**0.000**
RAP, mmHg	14 ± 6	8 ± 2	6 ± 1	4 ± 2	**0.000**
TPG, mmHg	40 ± 11	37 ± 11	39 ± 9	40 ± 11	0.550
CO, L/min	4.1 ± 1.1	4.6 ± 1.7	5.2 ± 1.7	4.9 ± 1.2	**0.004**
CI, L/min/m^2^	2.4 ± 0.5	2.6 ± 0.8	2.8 ± 0.9	2.8 ± 0.7	**0.001**
PVR, UW	11 ± 4	9 ± 4	8 ± 3	9 ± 3	**0.006**
Echocardiogram					
RVEF, %	66 ± 7	68 ± 7	66 ± 7	68 ± 7	0.499
RVSF, %	20 ± 6	27 ± 10	28 ± 9	31 ± 9	**0.000**
LVEF, %	29 ± 13	46 ± 17	36 ± 17	35 ± 5	0.108
RV pressure, mmHg	85 ± 23	80 ± 22	77 ± 23	81 ± 18	0.434

Bold values denote statistical significance at the *p* < 0.05 level.

**Table 2:** Baseline clinical features in two groups (cutoff value PAPi = 8.6) before and after matching. BMI, body mass index; CAD, coronary artery disease; PE, pulmonary embolism; DVT, deep venous thrombosis; NYHA, New York Heart Association; VKA, vitamin K antagonist; INR, international normalized ratio; CT, computed tomography; LVEF, left ventricular ejection fraction; RVSF, right ventricular shortening fraction; RVEF, right ventricular ejection fraction; RVp, right ventricular pressure; TR, tricuspid regurgitation; RAP, right atrial pressure; PAPs, systolic pulmonary artery pressure; PAPd, diastolic pulmonary artery pressure, PAPm, mean pulmonary artery pressure; PCWP, pulmonary capillary wedge pressure; CI, cardiac index; CO, cardiac output; TPG, transpulmonary gradient; PVR, pulmonary vascular resistance; WU, Wood units.

	Unmatched Cohort							Matched Cohort						
	Overall (*n* = 186)		PAPi ≤ 8.6 (*n* = 94)		PAPi > 8.6 (*n* = 92)			Overall (*n* = 134)		PAPi ≤ 8.6 (*n* = 67)		PAPi > 8.6 (*n* = 67)		
	n	%	n	%	n	%	*p*-Value	n	%	n	%	n	%	*p*-Value
Baseline features														
Age, years	58 ± 15		57 ± 17		59 ± 14		0.385	59 ± 15		60 ± 16		58 ± 15		0.536
Gender, female	116	63.4	59	62.8	57	62.0	0.909	79	59.0	38	56.7	41	61.2	0.598
BMI, kg/m^2^	26.1 ± 4.9		25.9 ± 4.9		26.2 ± 5.0		0.655	25.9 ± 4.7		25.7 ± 4.4		26.2 ± 4.9		0.569
Hypertension	88	47.3	43	45.7	45	48.9	0.665	64	47.8	30	44.8	34	50.7	0.489
Diabetes mellitus	7	3.8	5	5.3	2	2.2	0.260	3	2.2	1	1.5	2	3.0	0.500
Smoking history	60	32.3	29	30.9	31	33.7	0.678	41	30.6	21	31.3	20	29.9	0.851
Dyslipidemia	62	33.3	27	28.7	35	38.0	0.178	44	32.8	22	32.8	22	32.8	1.000
CAD	21	11.3	10	10.6	11	12.0	0.776	18	13.4	8	11.9	10	14.9	0.612
Lung disease	86	46.2	44	46.8	42	45.7	0.709	57	42.5	26	38.8	31	46.5	0.567
O_2_ therapy	50	26.9	28	29.8	22	23.9	0.365	35	26.1	20	29.9	15	22.4	0.298
Acute PE	112	60.2	51	54.3	61	66.3	0.075	82	61.2	39	58.2	43	64.2	0.478
DVT	84	45.2	39	41.5	45	48.9	0.303	66	49.3	31	46.3	35	52.2	0.601
Thrombophilia	56	30.1	24	25.5	32	34.8	0.153	43	32.1	16	23.9	27	40.3	**0.048**
NYHA class III-IV	126	67.7	69	73.4	57	62.0	0.102	92	68.7	48	71.6	44	65.7	0.515
REDO	11	5.9	6	6.4	5	5.4	0.784	9	6.7	4	6.0	5	7.5	0.500
VKA therapy	183	98.4	93	98.9	90	97.8	0.743	133	99.3	67	100.0	66	98.5	0.500
INR_pre	1.9 ± 0.7		1.9 ± 0.7		1.8 ± 0.7		0.219	1.8 ± 0.7		1.9 ± 0.6		1.8 ± 0.7		0.523
Vasodilators	127	68.3	62	66.0	65	70.7	0.358	89	66.4	43	64.2	46	68.6	0.583
CT scan involvement														
Pulmonary trunk	59	31.7	29	30.9	30	32.6	0.716	49	36.6	25	37.3	24	35.8	0.910
Lobar	110	59.1	53	56.4	57	61.9	0.381	87	65.9	40	59.7	47	70.2	0.205
Segmental	163	87.6	86	91.5	77	83.7	0.134	120	89.6	63	94.0	57	85.1	0.090
Subsegmental	105	56.5	60	63.8	45	48.9	0.057	74	55.2	43	64.2	31	46.3	**0.046**
Echocardiogram														
LVEF, %	67 ± 7		67 ± 7		67 ± 7		0.972	67 ± 8		67 ± 7		67 ± 8		0.793
RVSF, %	27 ± 10		24 ± 9		30 ± 9		**0.000**	27 ± 10		25 ± 10		30 ± 10		**0.009**
RVEF, %	35 ± 15		35 ± 16		36 ± 13		0.987	37 ± 16		37 ± 19		36 ± 13		0.842
RVp, mmHg	81 ± 22		82 ± 23		79 ± 21		0.355	79 ± 22		79 ± 22		79 ± 22		0.900
severe TR	51	27.4	33	35.1	18	19.6	**0.031**	36	26.9	21	31.3	15	22.4	0.286
Right heart catheterization														
RAP, mmHg	8 ± 5		11 ± 5		5 ± 2		**0.000**	8 ± 4		10 ± 4		5 ± 2		**0.000**
PAPs, mmHg	85 ± 18		84 ± 18		86 ± 19		0.383	83 ± 18		79 ± 16		86 + 20		**0.046**
PAPd, mmHg	26 ± 10		29 ± 12		23 ± 7		**0.000**	25 ± 8		27 ± 8		23 ± 7		**0.009**
PAPm, mmHg	49 ± 11		50 ± 12		48 ± 10		0.261	47 ± 10		46 ± 9		47 ± 11		0.591
PCWP, mmHg	10 ± 4		11 ± 5		8 ± 3		**0.000**	9 ± 3		10 ± 3		8 ± 3		**0.000**
TPG, mmHg	39 ± 11		39 ± 11		39 ± 10		0.628	38 ± 10		36 ± 10		39 ± 11		0.087
CO, L/min	4.7 ± 1.5		4.4 ± 1.5		5.0 ± 1.4		**0.002**	4.6 ± 1.4		4.4 ± 1.5		4.8 ± 1.3		0.072
CI, L/min/m^2^	2.6 ± 0.8		2.4 ± 0.7		2.8 ± 0.8		**0.000**	2.6 ± 0.7		2.4 ± 0.7		2.7 ± 0.6		**0.044**
PVR, UW	9.2 + 3.8		9.9 ± 4.2		8.5 ± 3.1		**0.011**	8.9 ± 3.3		9.0 ± 3.5		8.7 ± 3.2		0.600
PAPi, mmHg	10.3 + 7.2		5.6 ± 2.0		15.0 ± 7.4		**0.000**	10.4 ± 7.4		5.8 ± 1.9		15.0 ± 7.9		**0.000**

Bold values denote statistical significance at the *p* < 0.05 level.

**Table 3:** Postoperative outcomes in two groups (cutoff value PAPi = 8.6) before and after matching. CC, cross-clamp; CPB, cardiopulmonary bypass time; DHCA, deep hypothermic circulatory arrest; POD, postoperative day; RAP, right atrial pressure; PAPs, systolic pulmonary artery pressure; PAPd, diastolic pulmonary artery pressure, PAPm, mean pulmonary artery pressure; PCWP, pulmonary capillary wedge pressure; TPG, transpulmonary gradient; CO, cardiac output; CI, cardiac index; PVR, pulmonary vascular resistance; WU, Wood units; ECLS, extracorporeal life support; PND, permanent neurological deficit; RRT, renal replacement therapy; ICU, intensive care unit.

	Unmatched Cohort							Matched Cohort						
Early Outcomes	Overall (*n* = 186)		PAPI ≤ 8.6 (*n* = 94)		PAPI > 8.6 (*n* = 92)			Overall (*n* = 134)		PAPI ≤ 8.6 (*n* = 67)		PAPI > 8.6 (*n* = 67)		
	n	%	n	%	n	%	*p*-Value	n	%	n	%	n	%	*p*-Value
CC time, min	160 ± 47		169 ± 47		151 ± 47		**0.009**	159 ± 43		159 ± 38		160 ± 47		0.965
CPB time, min	284 ± 69		292 ± 77		276 ± 60		0.136	283 ± 62		284 ± 63		281 ± 62		0.795
DHCA time, min	30 ± 17		30 ± 18		30 ± 17		0.872	30 ± 18		30 ± 18		29 ± 18		0.770
Hemodynamics (1° POD)														
RAP, mmHg	7 ± 2		7 ± 3		7 ± 2		0.839	7 ± 2		7 ± 3		7 ± 2		0.862
PAPs, mmHg	40 ± 13		40 ± 14		40 ± 12		0.918	40 ± 13		40 ± 14		40 ± 11		0.896
PAPd, mmHg	18 ± 6		18 ± 7		18 ± 5		0.720	17 ± 6		18 ± 7		17 ± 5		0.526
PAPm, mmHg	25 ± 7		25 ± 8		25 ± 6		0.753	25 ± 7		25 ± 8		24 ± 6		0.654
PCWP, mmHg	9 ± 3		9 ± 3		9 ± 3		0.676	9 ± 3		9 ± 3		9 ± 4		0.422
TPG, mmHg	16 ± 7		16 ± 7		16 ± 7		0.841	16 ± 7		16 ± 8		15 ± 6		0.644
CO, L/min	4.5 ± 1.3		4.4 ± 1.3		4.6 ± 1.3		0.514	4.6 ± 1.3		4.5 ± 1.3		4.7 ± 1.2		0.433
CI, L/min/m^2^	2.6 ± 0.8		2.6 ± 0.7		2.6 ± 0.8		0.877	2.7 ± 0.8		2.6 ± 0.7		2.7 ± 0.9		0.829
PVR, UW	4.1 ± 2.9		4.1 ± 3.0		4.0 ± 2.7		0.843	3.7 ± 2.3		3.7 ± 2.1		3.7 ± 2.6		0.985
Rethoracotomy	25	13.4	14	14.9	11	12.0	0.577	21	15.7	12	17.9	9	13.4	0.476
ECLS	24	12.9	14	14.9	10	10.9	0.413	15	11.2	8	11.9	7	10.4	0.784
Support time, days	10 ± 8		9 ± 8		10 ± 8		0.842	9 ± 8		7 ± 8		12 ± 8		0.248
Successful weaning	14	7.5	6	6.4	8	8.7	**0.080**	8	6.0	2	25.0	6	9.0	**0.032**
PND	2	1.1	1	1.1	1	1.1	0.794	2	1.5	1	1.5	1	1.5	0.752
Prolonged ventilation (>48 h)	54	29.0	34	36.2	22	23.9	**0.083**	38	28.4	23	34.3	15	22.4	0.123
Pneumonia	33	17.7	23	24.5	10	10.9	**0.015**	25	18.7	16	23.9	9	13.4	0.121
Tracheostomy	14	7.5	6	6.4	8	8.7	0.564	11	8.2	5	7.5	6	9.0	0.753
Sepsis	16	8.6	9	9.6	7	7.6	0.617	10	7.5	6	9.0	4	6.0	0.511
Permanent RRT	6	3.2	3	3.2	3	3.3	0.648	5	3.7	3	4.5	2	3.0	0.500
ICU stay, days	8 ± 9		8 ± 9		8 ± 9		0.863	8 ± 9		8 ± 8		9 ± 10		0.689
30-day mortality	16	8.6	10	10.6	6	6.5	0.317	9	6.7	7	10.4	2	3.0	0.082
90-day mortality	18	9.6	12	12.7	6	6.5	0.150	9	6.7	7	10.4	2	3.0	0.082

Bold values denote statistical significance at the *p* < 0.05 level.

## Data Availability

The data supporting the findings of this study are available from the corresponding author upon reasonable request.
